# ﻿*Amalophyllonmiraculum* (Gesneriaceae), an exceptionally small lithophilous new species from the western Andean slopes of Ecuador

**DOI:** 10.3897/phytokeys.242.118069

**Published:** 2024-06-11

**Authors:** John L. Clark, Andrea Fernández, J. Nicolás Zapata, Camilo Restrepo-Villarroel, Dawson M. White, Nigel C. A. Pitman

**Affiliations:** 1 Marie Selby Botanical Gardens, 1534 Mound St., Sarasota, FL 34236, USA Marie Selby Botanical Gardens Sarasota United States of America; 2 Herbario QCA, Escuela de Ciencias Biológicas, Pontificia Universidad Católica de Ecuador, Av. 12 de Octubre 1076 y Roca, Apartado17-01-2184, Quito, Ecuador Pontificia Universidad Católica de Ecuador Quito Ecuador; 3 Estación de Biodiversidad Tiputini, Colegio de Ciencias Biológicas y Ambientales, Universidad San Francisco de Quito-USFQ, Quito, Ecuador Universidad San Francisco de Quito Quito Ecuador; 4 Harvard University Herbaria, 22 Divinity Avenue, Cambridge, MA 02138, USA Harvard University Herbaria Cambridge United States of America; 5 Collections, Conservation and Research, Field Museum of Natural History, 1400 S. Du Sable Lake Shore Drive, Chicago, IL 60605, USA Field Museum of Natural History Chicago United States of America

**Keywords:** Andes, *Amalophyllon*, Centinela, Chocó, Conservation, Ecuador, endemic, lithophyte, Montañas de Ila

## Abstract

Recent exploratory field expeditions to the western slopes of the Ecuadorian Andes resulted in the discovery of a new species of *Amalophyllon* (Gesneriaceae). *Amalophyllonmiraculum* J.L.Clark, **sp. nov.** is described from two localities in the Centinela region in the Santo Domingo de los Tsáchilas province. The new species is differentiated from congeners by the pendent habit, basal rosette of leaves, leaf blades with deeply serrate margins, and miniature size. Based on IUCN guidelines, a preliminary conservation status is assigned as Critically Endangered (CR).

## Abstract (es)

Recientes expediciones exploratorias de campo a las laderas occidentales de los Andes ecuatorianos dieron como resultado el descubrimiento de una nueva especie de *Amalophyllon* (Gesneriaceae). *Amalophyllonmiraculum* J.L.Clark, **sp. nov.** se describe de dos localidades de la región de Centinela en la provincia de Santo Domingo de los Tsáchilas. La nueva especie se diferencia de otros congéneres por el hábito colgante, la roseta basal de las hojas, las láminas foliares con márgenes profundamente aserrados y su tamaño en miniatura. Según las directrices de la UICN, se le asigna el estado de conservación preliminar de En Peligro Crítico (CR).

## ﻿Introduction

The flowering plant family Gesneriaceae is in the order Lamiales and comprises 3400+ species in 150+ genera (Weber 2004; Weber et al. 2013). The family is divided into three strongly supported monophyletic subfamilies (Ogutcen et al. 2021) and seven tribes (Weber et al. 2013, 2020). The majority of New World members are in the subfamily Gesnerioideae and are represented by 1200+ species and 77 genera (Clark et al. 2020; GRC 2024). *Amalophyllon* Brandegee is classified in the tribe Gesnerieae and subtribe Gloxiniinae (Weber et al. 2013, 2020).

*Amalophyllon* is a genus of terrestrial or lithophytic herbs distributed from Mexico (Chiapas) through Central America to Venezuela, Colombia, Ecuador, and northern Peru. The presence of subrotate to rotate white corollas defines the following three currently recognized genera from the subtribe Gloxiniinae G.Don: *Amalophyllon*, *Niphaea*, and *Phinaea*. Phylogenetic studies based on molecular sequence data (Smith et al. 2004; Roalson et al. 2005; Roalson et al. 2008; Clark et al. 2011) strongly support the independent origins of subrotate to rotate corollas from recent ancestors with corollas that are bilaterally symmetrical and tubular. The similarity of floral structures in these three clades is likely a convergence that is correlated with vibrational or a “buzz” pollination syndrome (Wiehler 2002).

**Figure 1.**
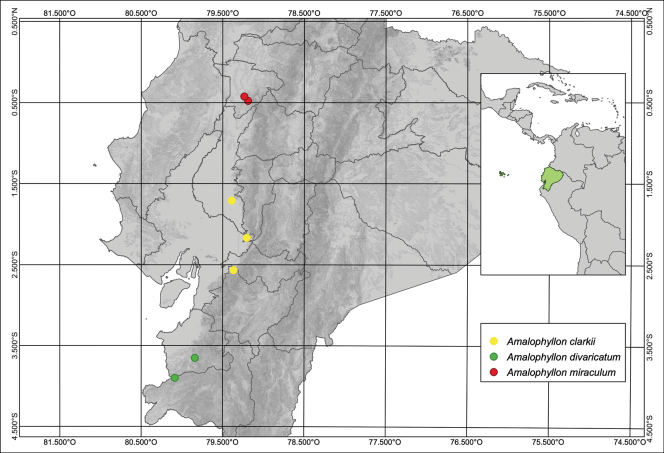
Distribution map of *Amalophyllon* in Ecuador. Localities with yellow circles =*Amalophyllonclarkii* Boggan & L.E.Skog, green circles =*A.divaricatum* (Poepp.) Boggan, L.E.Skog & Roalson, and red circles =*A.miraculum* J.L.Clark.

*Amalophyllon* was initially recognized as a monotypic genus in the Scrophulariaceae (Brandegee 1914). More recently, the name was applied to Gesneriaceae by Boggan et al. (2008) based on phylogenetic studies that strongly supported a clade that included several previously recognized species of *Phinaea*. Thus, *Amalophyllon* was expanded by Boggan et al. (2008) to include 13 species. The description of *Amalophyllonmiraculum* increases the total to 14 species of *Amalophyllon*. There are currently two species of *Amalophyllon* in Ecuador and the addition of *A.miraculum* increases the total to three species (Fig. 1). *Amalophyllondivaricatum* is known from two populations in southern Ecuador (El Oro and Loja) and at least four populations in Peru (Huánuco, Junín, San Martín, Ucayali). *Amalophyllonclarkii* Boggan & L.E.Skog is endemic to western Ecuador. The type locality of *A.clarkii* is in Azuay, near the provincial border of Guayas in the Bosque Protector Molleturo Mullopungo (Fig. 1). Additional populations of *A.clarkii* are in the Ecuadorian provinces of Guayas and Los Ríos (Fig. 1).

The genera *Amalophyllon*, *Niphaea*, and *Phinaea* were recently circumscribed based on phylogenetic studies (Smith et al. 2004; Roalson et al. 2005; Roalson et al. 2008; Clark et al. 2011). A summary of morphological differences between *Amalophyllon*, *Niphaea*, and *Phinaea* is provided in Boggan et al. (2008). There are floral characters that differentiate *Niphaea*, but there are only fruit characters that separate *Phinaea* from *Amalophyllon*. In addition, a fourth clade of rotate flowers is represented by “*Phinaea” pulchella* (Griseb.) C.V.Morton from Cuba (Clark et al. 2011). *Amalophyllon* differs from *Phinaea* by fruit characters outlined in Boggan et al. (2008). The capsules of *Phinaea* are subtended by erect pedicels. In contrast, the pedicel posture in *Amalophyllon* is usually curved. The seeds in *Phinaea* are typically sticky and adhere to the fruit valves, whereas the seeds fall freely from the capsules of *Amalophyllon*.

## ﻿Materials and methods

Plants were vouchered and photographed during two field expeditions to the western Andes of Ecuador in 2022 (Clark 2022). Specimens were deposited in the following herbaria: Pontificia Universidad Católica del Ecuador (QCA), Marie Selby Botanical Gardens (SEL), United States National Herbarium (US), New York Botanical Garden (NY), and Missouri Botanical Garden (MO). Digital images of live specimens were taken in the field using a Nikon D100 DSLR with a Nikon 105 mm lens and a Nikon SB-29s ring flash. Morphological observations and measurements were made from live collections, alcohol-preserved material, and digital images using the software program *ImageJ* (Schneider et al. 2012).

We assessed the extinction risk of *Amalophyllonmiraculum* following the IUCN Red List Categories and Criteria and guidelines of the IUCN (2022). We considered observations, collection localities and population estimates from fieldwork. We refrained from calculating extent of occurrence (EOO) because of the limited number of known populations. The area of occupancy (AOO) was calculated using the software program *GeoCAT* (Bachman et al. 2011) with the default setting of a 4 km^2^ grid.

## ﻿Taxonomic treatment

### ﻿ Amalophyllon miraculum

Taxon classificationPlantaeLamialesGesneriaceae

J.L.Clark sp. nov.

48FEC23A-4208-53AD-96F1-C5E2CEEBAB34

urn:lsid:ipni.org:names:77343140-1

Fig. 2

#### Type.

Ecuador. Santo Domingo de los Tsáchilas: cantón Santo Domingo, parroquia El Esfuerzo, El Respaldo, 3.5 km east of Segundo Respaldo, finca de Paul Henry, 0°25'25.8"S, 79°14'7.4"W, 672 m alt., 19 Mar 2022, *J.L. Clark, X. Cornejo, P. Henry & C. Restrepo-Villarroel 16634* (holotype: QCA; isotypes: G, MO, NY, SEL, US).

#### Diagnosis.

Similar to *Amalophyllonclarkii*, differing in larger and broadly ovate leaves in *A.clarkii* (>8 cm long) vs. smaller elongate to lanceolate leaf blades in *A.miraculum* (< 6 cm long); calyx lobes elongate in *A.clarkii* vs. broadly oblong in *A.miraculum*; and abaxial leaf surface green with purple venation in *A.clarkii* vs. uniformly dark purple in *A.miraculum*.

#### Description.

Lithophytic herb with scaly rhizomes; stem short; pendent to horizontal with leaves in a basal rosette. ***Leaves*** opposite, subequal; petiole glabrous to sparsely pubescent, 2–5 mm long; blade membranous, fragile when dried, oblong to lanceolate, 1.5–5.0 cm long, 1–2 cm wide, with 6–9 pairs of lateral veins, margins deeply serrate, bright green with dark green venation on adaxial surface, dark purple on abaxial surface, apex acute. ***Inflorescence*** reduced to a single axillary flower (without peduncles), usually produced at the apex of leaves or axis of clustered leaves, with 1–3 flowers per axil, inflorescence bracts absent; pedicels slender, curved, 1.5–2.0 cm long; calyx lobes 5, uniformly green, subequal, broadly oblong, nearly free, entire, rounded at apex, ca. 2.0 mm long × 1.0 mm wide; corolla lobes 5, fused at base for 1–2 mm forming a shallow tube, tube light red, lobes uniformly white, lobes entire, subequal, spreading broadly during anthesis, apices rounded, corolla lobes broadly ovate, ca. 2.5 mm long × 2.5 mm wide, glabrous inside and outside; stamens 4, adnate to the base of the corolla tube, filaments yellow, ca. 0.5 mm long, glabrous; nectary absent; ovary nearly superior, subglobose, glabrous, ca. 1 mm long and wide, style ca. 2 mm long, curved, glabrous, stigma capitate. ***Fruits*** not observed.

**Figure 2.**
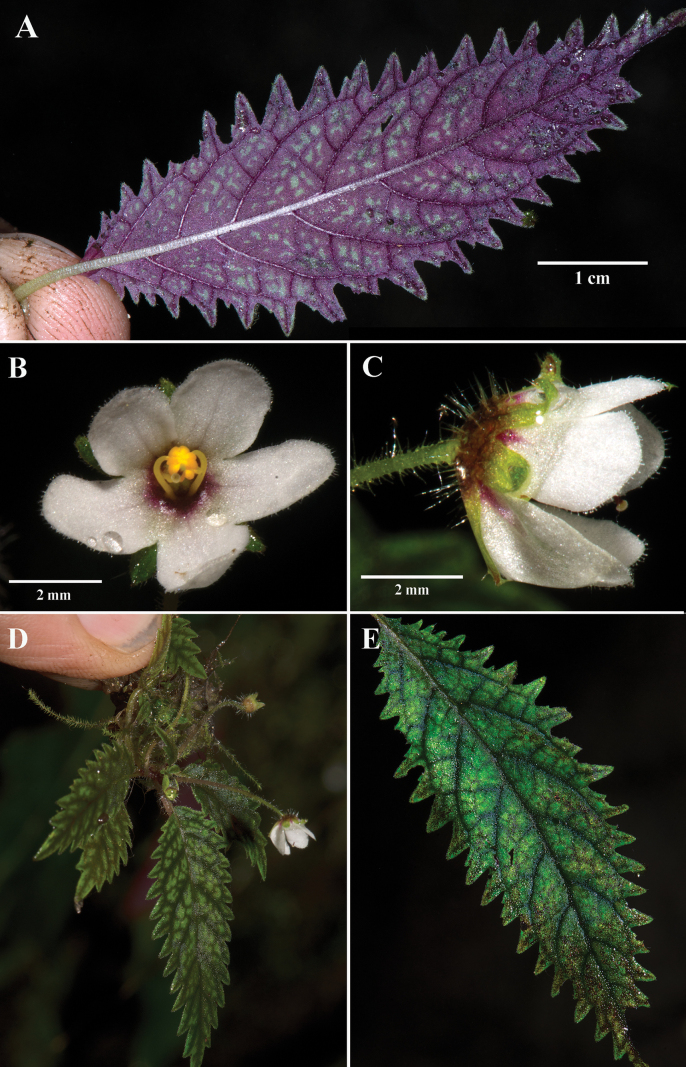
*Amalophyllonmiraculum* J.L.Clark **A** abaxial view of leaf **B** front view of flower **C** lateral view of flower **D** pendent habit featuring rosette of leaves **E** adaxial view of leaf. (**A, E** from *J.L. Clark et al. 16805*; **B, C, D** from *J.L. Clark et al. 16634*). Photos by J.L. Clark.

#### Phenology.

Flowering in March and July. Fruits not observed.

#### Etymology.

The specific epithet reflects the extraordinary and unexpected persistence of remnant forest patches of an area broadly defined as “Centinela” (see next section). Dodson and Gentry (1991) popularized this legendary biodiversity hotspot and brought it to prominence when they reported a mass extinction of plant species from this region. Many of the presumed “extinct” species were recently documented, including *Gasteranthusextinctus* L.E.Skog & L.P.Kvist (Gesneriaceae) (Pitman et al. 2022). *Amalophyllonmiraculum* is sympatric with *Gasteranthusextinctus*. The presence of several critically endangered species and the recent discovery and description of new species from Centinela represent a miraculous discovery that has shattered a prevailing assumption that the once-thought-lost biodiversity of Centinela had vanished entirely. The heroic efforts of local landowners who maintained small patches of forests (usually surrounding waterfalls) were instrumental in conserving remnant forest fragments. Also crucial are current conservation initiatives by foundations and academic institutions such as the Ecuadorian conservation NGO Fundación de Conservación Jocotoco and the Jardín Botánico Padre Julio Marrero (JBJM) of the Pontificia Universidad Católica del Ecuador in the nearby city of Santo Domingo.

#### Distribution and preliminary assessment of conservation status.

*Amalophyllonmiraculum* has been collected in Ecuador’s western Andean slopes in the Santo Domingo de los Tsáchilas province. The only two known subpopulations are in small patches of forest surrounded by large swaths of deforested agricultural landscapes. The forest patch at the Paul Henry farm is approximately 10 hectares and is located in the northernmost part of the Montañas de Ila range in Recinto Milton Murillo. The southern forest patch in the Bosques y Cascadas Las Rocas private reserve is approximately 50 hectares and lies in the intermontane area between the Andean Cordillera and the northern Montañas de Ila (Fig. 1). These patches are approximately 8 km from each other. The current landowners (Paul Henry and Eduardo Díaz C.) are committed to preserving the forest fragments on their property, but broader efforts are urgently needed by governmental and non-governmental agencies to protect these and other nearby forest fragments. The GeoCAT calculated AOO is 8 km^2^. *Amalophyllonmiraculum* is preliminarily assessed as Critically Endangered (CR) based on a limited area of occupancy (IUCN criterion B1 where AOO <10 km^2^) and the severely fragmented forests (B2a) and ongoing decline of the Centinela forests in western Ecuador (B2bi, ii, iii, iv). Intact forests in the Centinela region are mostly reduced to small (<10 hectares) fragments. Extensive deforestation in western Ecuador, especially Centinela, has resulted in an alarming habitat loss. The area was popularized by E.O. Wilson’s (1992) term as the ‘Centinelan extinction’ because of initial reports of wide-scale plant extinctions by Dodson and Gentry (1991). One of the presumed extinctions was *Gasteranthusextinctus* L.E.Skog & L.P. Kvist (Gesneriaceae), which was recently documented in more than five forest fragments (Pitman et al. 2022). The rediscovery resulted in a re-evaluation of its IUCN assessment from Critically Endangered (CR) to Endangered (EN). We conducted five field expeditions between 2021 and 2023 and located the only two currently known populations of *Amalophyllonmiraculum*, which is sympatric with the more widespread *Gasteranthusextinctus*. The only documented populations of *Amalophyllonmiraculum* are inside privately protected areas surrounded by agriculture in unprotected parts of the Santo Domingo de los Tsáchilas province. Effective conservation of this and the other endemic species of the Centinela region will require constant vigilance.

Locating current and future populations of *Amalophyllonmiraculum* is a major challenge because of their small size, ephemeral flowers, and camouflaged foliage on wet moss-covered rock faces. For example, authors Fernández and Zapata recently (April 2024) searched forests in the type locality in Paul Henry’s farm but did not locate extant populations. Likewise, it will require targeted and careful searching to document and locate this elusive species.

#### Comments.

**Figure 3.**
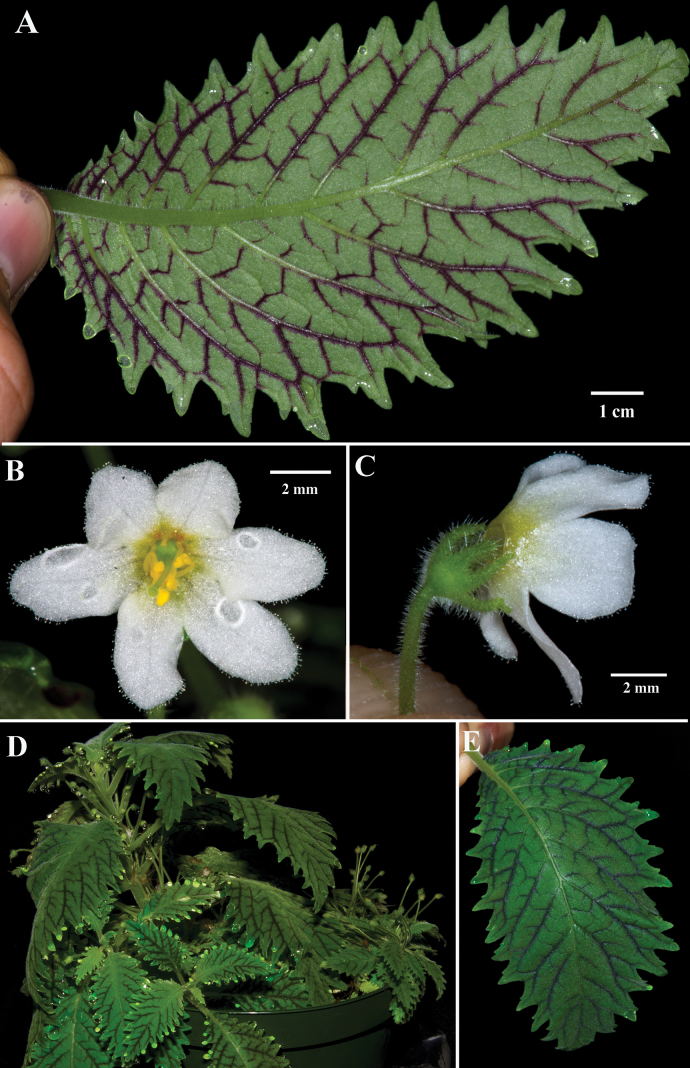
*Amalophyllonclarkii* Boggan & L.E.Skog **A** abaxial view of leaf **B** front view of flower **C** lateral view of flower **D** erect habit featuring evenly spaced and rosette-forming leaves **E** adaxial view of leaf. (**A–E** from *J.L. Clark 13101*). Photos by J.L. Clark.

Most *Amalophyllon* have leaf margins that are serrate to crenate. *Amalophyllonmiraculum* and *A.clarkii* (Fig. 2A) are differentiated from other congeners by the presence of deeply serrate to biserrate leaf margins (Figs 2A, 3A). The leaf blades of *A.clarkii* are broadly ovate and nearly 8 cm long (Fig. 2A). In contrast, the leaves of *A.miraculum* are never more than 6 cm long (Fig. 2A). The calyx lobes in *A.clarkii* are elongate and narrow (Fig. 3C) vs. broadly oblong in *A.miraculum* (Fig. 2C). Both *Amalophyllonclarkii* and *A.miraculum* share a lithophytic habit but differ in their habitat and posture. Populations of *A.clarkii* were observed growing erect on a rock in the understory of a shaded forest without direct moisture. Populations of *A.miraculum* are pendent and have only been observed on wet rocks in streams or where mist is persistent. It was common to locate populations of 10–20 individuals of *A.miraculum* on wet areas of rock faces and no populations on adjacent dry areas, even when mosses and ferns were shared between the two microhabitats. The rosette-forming individuals of *A.miraculum* were often pendent. In contrast, populations of *A.clarkii* are either rosette-forming or with elongate erect shoots, but usually erect. There are always five corolla lobes in *Amalophyllonmiraculum*. In contrast, the number of corolla lobes in *A.clarkii* is usually five, but occasionally six (Fig. 3B).

*Amalophyllonmiraculum* and *A.clarkii* are geographically isolated. *Amalophyllonmiraculum* is a narrow endemic from the northern lowlands of the western Andes of Ecuador in the province of Santo Domingo de los Tsáchilas (Fig. 1). Populations of *A.clarkii* are mainly from the southern lowlands of western Ecuador (Azuay, Guayas, and Los Ríos). One disjunct population of *A.clarkii* was reported in Boggan et al. (2008) from a unicate collection by Alexander Hirtz from the northern province of Esmeraldas. The collection by Hirtz (*A. Hirtz 3629* - SEL) could not be located or verified and is therefore not included in the distribution map (Fig. 1).

#### Additional specimen examined.

Ecuador. Santo Domingo de los Tsáchilas: cantón Santo Domingo, parroquia Polanco, sector Bolo Alto, Bosques y Cascadas Las Rocas, propiedad de Eduardo Díaz, near waterfall of the Bolo watershed, 0°28'38.1"S, 79°11'22.4"W, 560–600 m alt., 19 Mar 2022, *J.L. Clark, L. Hooge, C. Restrepo-Villarroel, R. Clark & E. Muñoz 16805* (MO, NY, QCA, SEL, US).

## Supplementary Material

XML Treatment for Amalophyllon miraculum

## ﻿Additional information

### Author ORCIDs

John L. Clark https://orcid.org/0000-0002-1414-6380

J. Nicolás Zapata https://orcid.org/0000-0002-2863-6524

Camilo Restrepo-Villarroel https://orcid.org/0009-0000-2474-9460

Dawson M. White https://orcid.org/0000-0002-0670-9390

Nigel C. A. Pitman https://orcid.org/0000-0002-9211-2880

### Data availability

All of the data that support the findings of this study are available in the main text.
